# The impact of random frequency-dependent mutations on the average population fitness

**DOI:** 10.1186/1471-2148-12-160

**Published:** 2012-08-30

**Authors:** Weini Huang, Benjamin Werner, Arne Traulsen

**Affiliations:** 1 Evolutionary Theory Group, Max Planck Institute for Evolutionary Biology Plön, 24306 Plön, August-Thienemann-Straße 2, Germany

## Abstract

**Background:**

In addition to selection, the process of evolution is accompanied by stochastic effects, such as changing environmental conditions, genetic drift and mutations. Commonly it is believed that without genetic drift, advantageous mutations quickly fixate in a halpoid population due to strong selection and lead to a continuous increase of the average fitness. This conclusion is based on the assumption of constant fitness. However, for frequency dependent fitness, where the fitness of an individual depends on the interactions with other individuals in the population, this does not hold.

**Results:**

We propose a mathematical model that allows to understand the consequences of random frequency dependent mutations on the dynamics of an infinite large population. The frequencies of different types change according to the replicator equations and the fitness of a mutant is random and frequency dependent. To capture the interactions of different types, we employ a payoff matrix of variable size and thus are able to accommodate an arbitrary number of mutations. We assume that at most one mutant type arises at a time. The payoff entries to describe the mutant type are random variables obeying a probability distribution which is related to the fitness of the parent type.

**Conclusions:**

We show that a random mutant can decrease the average fitness under frequency dependent selection, based on analytical results for two types and simulations for *n* types. Interestingly, in the case of at most two types the probabilities to increase or decrease the average fitness are independent of the concrete probability density function. Instead, they only depend on the probability that the payoff entries of the mutant are larger than the payoff entries of the parent type.

## Background

Mutations provide a continuous source of variation in natural populations, on which natural selection can act. When fitness is assumed to be constant, only those mutations with higher fitness values will be fixed in a haploid population under strong selection and negligible random drift. Thus, the average fitness of the population would monotonically increase in evolutionary time. There have been numerous hypotheses why this is not what is observed in nature: for instance, environmental changes require new adaptions [1,2] or coevolution can imply continuous adaptation without increasing the average fitness [3-5]. However, these are not aspects that we intend to include here. Instead, we focus on a haploid population in a constant environment, and explore frequency dependent fitness, which can be described by evolutionary game theory [6-11]. In this framework, the fitness of a type depends on the frequencies of other types of individuals in the population. We address the very general question of how the average fitness changes when it is driven by random mutations under frequency dependent selection.

The fitness effects of new mutations have gained significant attention both in experimental research and theoretical work [12,13]. In experiments, the distribution of fitness effects depends on several aspect of the experimental setup, e.g. how well adapted the organism is to the environment and whether only single mutants or also double mutants (mutants differing from the wild type by two mutations) are considered. Different shaped distributions were proposed to capture the fitness distributions of random mutants under constant selection [14-17]. The concrete shape of fitness distributions of spontaneous mutations varies between species and even within the same species on different parts of DNA [18]. Although no common conclusion on this has been obtained yet – and a universal fitness distribution may as well not exist – it is often possible to estimate some general properties, such as the proportion of advantageous mutations and the mean value of the fitness of the mutations [19,20].

The concept of random distributed and frequency dependent fitness of mutations can be addressed by evolutionary game theory [21], which considers evolutionary processes under frequency dependent selection [22]. In this framework, a population of interacting individuals is considered. In the simplest case of linear frequency dependence, the interactions of different types of individuals are captured by a payoff matrix for a game. Those types which are more successful in the game will have a higher reproduction rate. We introduce a payoff matrix with variable size to capture mutations and extinctions. The new payoff entries introduced by mutations are independently drawn from a probability distribution, which corresponds to the concept of randomly distributed fitness. By tracking the dynamics of the payoff matrix and the compositions of the population, we are able to investigate several aspects of an evolving system, such as the average fitness changes of the population, the impact of the fitness distribution on these changes and the expected level of diversity.

## Results

### Dynamics for populations with two types

Let us start with a population of a resident wild type (*R*) and a mutant type (*M*). Suppose the fitness of a wild type in a homogenous population is *d*. For constant selection, the fitness distribution of a mutant is simply a one dimensional distribution around *d*. For frequency dependent selection, the fitness of a mutant must be defined based on more than a single number. We can write it as an evolutionary game based on a 2×2 payoff matrix with three new payoff entries, *a*, *b* and *c*

When a mutant and a wild type interact, the mutant obtains fitness *a*, and the wild type obtains *c*. When a mutant meets another mutant, it obtains *b*. Following the concept of randomly distributed fitness of mutations, the entries *a*, *b* and *c* are defined as random variables. We assume that *a*, *b* and *c* independently follow the same probability distribution given by a probability density function *f*(*x*). While this is the simplest possibility, it may be more realistic to assume correlations between the payoff entries characterizing each type, i.e. between *a* and *b* as well as between *c* and *d* (see below, section Games with equal gains from switching). However, in the extreme case of *a *=* b* and *c *=* d*, this would recover the case of constant selection, so we expect that such correlations would lead to results intermediate between constant and frequency dependent selection. We discuss how this distribution affects the changes in the average fitness during the evolutionary process. It turns out, the probability $\theta =\int_{d}^{\infty }dx\;f(x)$ that a payoff entry is larger than the fitness of the wild type (the parent type in the case of *n* types) *d*, is of particular interest and determines the change in the average fitness. Remarkably, all other aspects of the fitness distribution turn out to be irrelevant for this observable.

The dynamics of evolving populations shows stochastic fluctuations when selection is weak and when populations are small. In addition, stochasticity can arise based on environmental changes or stochastic effects due to mutations. As we are interested in the effects of frequency dependent selection, we only consider stochasticity arising from random frequency dependent mutations and use the replicator equations to model evolutionary dynamics. The frequency of a certain type changes deterministically according to the difference of its own fitness to the average fitness in the population.

Suppose *x* is the frequency of the mutant type and 1 −* x *the frequency of the wild type, respectively. We can define the fitness of the mutant type, *W*_1_, and the fitness of the wild type, *W*_2_, as

(1) $$\begin{array}{l} W_{1}=\mathrm{ax}+b(1-x),\\ W_{2}=\mathrm{cx}+d(1-x), \end{array}$$

where *a*, *b*, *c*, and *d* are the entries in the payoff matrix. The average fitness of the population $\bar{W}$ is given by

(2) $$\bar{W}=xW_{1}+\left(1-x\right)W_{2}.$$

If the fitness of the mutant type is larger than the average fitness, its frequency will increase. If the fitness of the mutant type is below the average fitness, its frequency will decrease. We follow the usual assumption that the change of the frequency of the mutant type is given by the replicator equation [23-25]

(3) $$\dot{x}=x\left(W_{1}-\bar{W}\right)=x\left(1-x\right)\left(W_{1}-W_{2}\right).$$

The change of the wild type frequency follows immediately as $-\dot{x}$. This dynamics is fully determined by the entries of the payoff matrix. Different constellations of the payoff entries cause different dynamical patterns. In the following, we discuss all generic cases of two-type interactions and how the average fitness of the population changes under the different situations.

First, we analyze the case where the mutant has higher fitness than the wild type for all frequencies *x*. This is the case for *a *>* c* and *b *>* d*. The wild type goes extinct and the mutant type will be fixed in the population. Thus, the average fitness $\bar{W}$ in the new equilibrium *x *= 1 is given by the payoff entry of the mutant type interacting with itself, *a*. We are interested in the probability, that the fitness of the population is increased after the fixation of the mutant. This becomes a conditional probability of *a *>* d* given that *a *>* c* and *b *>* d*. Applying Bayes Rule, this can be expressed as

(4) $$\begin{array}{ll} \;p(\bar{W}(1)>d|a>c,b>d) & =p(a>d|a>c,b>d)\;\\ & =\frac{p(a>d,a>c,b>d)}{p(a>c,b>d)}\;\\ & =\frac{p(a>d,a>c)}{p(a>c)}. \end{array}$$

We assume that the random variables *a*, *b* and *c* are independently derived from the same probability distribution. Hence, *b* does not depend on *a* or on *c*. Thus, the probability of *b *>* d* is independent from the probability that *a *>* d*, which is used in Eq. (4). Since *a* and *c* are sampled from the same distribution, we have *p*(*a *>* c*) = 1/2 in the denominator. For the numerator, we have

(5) $$\begin{array}{ll} \;p(a>d,a>c) & =\int_{d}^{\infty }da\int_{-\infty }^{a}dc\;f(c)f(a)\;\\ & =\int_{d}^{\infty }da\;F(a)\;F^{\prime }(a)\\ & =\frac{1}{2}-\frac{F{\left(d\right)}^{2}}{2}, \end{array}$$

where *F*(*x*) is the cumulative distribution function of a random variable with probability density function *f*(*x*). The probability that one of the new payoff entries *a,b,c* is greater than the wild type fitness *d* is $\theta =\int_{d}^{\infty }dx\;f(x)=1-F(d)$. Using this expression in Eq. (5), we arrive at

(6) $$p(\bar{W}(1)>d|a>c,b>d)=2\theta -\theta^{2}.$$

Strikingly, this only depends on *θ*, and is independent of the concrete choice of the probability density function *f*(*x*). In population genetics, beneficial mutation rates are measured based on the concept of constant fitness, where the fitness of the mutant and the fitness of the wild type are both constant numbers. However, if we consider frequency dependent fitness, a new parameter is needed to represent the proportion of beneficial mutations. One option arising from our approach is to compare the payoff value of the mutant with the payoff value of the wild type when they are confronted by the same opponent. Since *θ *is the probability that the new payoff value of the mutant is larger than the wild type’s payoff *d*, it corresponds to the probability that a mutation is beneficial under the constant selection scenario. If *θ* can be measured, the probability that the average fitness is increased by a random mutant is independent of the payoff distribution according to Eq. (5). But different choices of probability density functions *f*(*x*) will result in different values of *θ*, thus leading to different probabilities to increase the average fitness.

Next, we assume that a mutant type occurs with lower fitness than the wild type. With frequency dependence, there are two situations for such a mutant type. The mutant type can either have lower fitness than the wild type for all frequencies, or it can have a lower fitness only for small frequencies. In both cases, the mutant will go extinct and the average fitness will remain unchanged, since a mutant type is supposed to arise with a small amount.

Finally, a mutant type could be initially advantageous compared to wild types, but turn to be disadvantageous when it has reached a certain frequency. This occurs for *a *<* c* and *b *>* d*. In this case neither the wild type nor the mutant type can take over the population, but there exists a mixed equilibrium consisting of the mutant type at a frequency $x^{*}=\frac{b-d}{b-d-a+c}$ and the wild type at a frequency 1 −* x*^∗^. In this coexistence equilibrium, the fitness of the wild type subpopulation is equal to the fitness of the mutant type subpopulation. The average fitness of the system in the equilibrium is given by

(7) $$\begin{array}{l} \bar{W}(x^{*})=ax^{*}+b(1-x^{*})=\frac{\mathrm{bc}-\mathrm{ad}}{b-d-a+c}. \end{array}$$

Again, we ask for the probability of having a coexistence game that increases the average fitness. This is the conditional probability that $\bar{W}\left(x^{*}\right)>d$ given that *a *<* c *and *b *>* d*, which can be written as

(8) $$\begin{array}{ll} & p(\bar{W}(x^{*})>d|a<c,b>d)\\ & =p\left(\left(b-d\right)\left(c-d\right)>0|a<c,b-d>0\right)\\ & =p\left(c>d|a<c\right)\\ & =\frac{p\left(c>a,c>d\right)}{p\left(c>a\right)} \end{array}$$

This is identical to Eq. (4) if one exchanges *a *⇔* c*. Since *a* and *c* have the same distribution, we recover the result from Eq. (6),

(9) $$p(\bar{W}(x^{*})>d|a<c,b>d)=2\;\theta -\theta^{2}.$$

In other words, the probability to increase fitness is the same in a coexistence game as in a game where the mutant dominates the wild type.

Let us now combine the results and consider the changes of the average fitness over all types of interactions. The probability to increase the fitness due to a new mutation is given by

(10) $$\begin{array}{ll} \;p(\bar{W}>d) & =\underset{2\theta -\theta^{2}}{\underset{︸}{p(\bar{W}>d|a>c,b>d)}}\underset{\frac{\theta }{2}}{\underset{︸}{p(a>c,b>d)}}\\ & \;+\underset{2\theta -\theta^{2}}{\underset{︸}{p(\bar{W}>d|a<c,b>d)}}\;\underset{\frac{\theta }{2}}{\underset{︸}{p(a<c,b>d)}}\\ & \;+\underset{0}{\underset{︸}{p(\bar{W}>d|b<d)}}\;\underset{1-\theta }{\underset{︸}{p(b<d)}}\\ & \;=2\;\theta^{2}-\theta^{3} \end{array}$$

In a similar manner, we can calculate the probability to decrease the average fitness due to a new mutation. When the mutant dominates the wild type, the average fitness may still decrease. This is exactly what happens in the Prisoner’s Dilemma [26,27]. Equivalently to the calculation above, we have

(11) $$\begin{array}{ll} \;p(\bar{W}(1)<d|a>c,b>d) & =\frac{p(a<d,a>c,b>d)}{p(a>c,b>d)}\;\\ & =\frac{p(a<d,a>c)}{p(a>c)}\\ & ={\left(1-\theta \right)}^{2}. \end{array}$$

For the probability to decrease the average fitness in a coexistence game, we find

(12) $$\begin{array}{ll} & p(\bar{W}(x^{*})<d|a<c,b>d)={\left(1-\theta \right)}^{2}. \end{array}$$

Thus, using a calculation similar to Eq. (10), the overall probability to decrease the average fitness is given by

(13) $$\begin{array}{l} p(\bar{W}<d)=\theta -2\theta^{2}+\theta^{3}. \end{array}$$

Also the probability to maintain a constant average fitness can be calculated in this way. For continuous fitness distributions, there are no strictly neutral mutations. As the fitness of the wild type is a specific value of the continuous random variable, the probability of having a strict neutral mutation, the fitness of which is equal to the fitness of the wild type, is 0. Thus, the average fitness is only maintained when the mutant goes extinct, which occurs with probability

(14) $$\begin{array}{l} p(\bar{W}(0)=d)=p(b<d)=1-\theta . \end{array}$$

**Figure 1:**
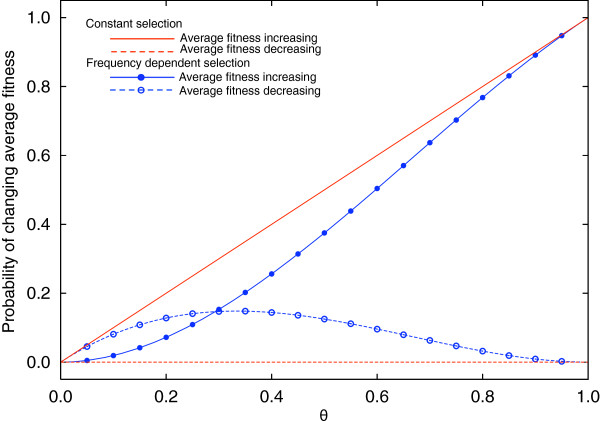
**Probability of increasing or decreasing the average fitness in the new equilibrium after one mutation event in an initial homogenous population. ***θ *is the probability that a random payoff entry of the mutant, *a*, *b* or *c* is larger than wild type initial fitness *d*. Blue symbols and lines are simulation and analytical results under frequency dependent selection (average over 10^6 ^runs). Red lines are analytical results under constant selection. For constant selection, the average fitness either increases or is unchanged by a new mutation, where the fraction of mutants that increases fitness is determined by *θ*. However, under frequency dependent selection, the average fitness of the population in the new equilibrium after a mutation can also decrease. The probability to increase, decrease the average fitness or maintain the same average fitness, depends on *θ*, for $\theta >\frac{\sqrt{2}-1}{\sqrt{2}}$ the probability to increase the average fitness in the new equlibrium is larger than the probability to decrease it.

We discussed the changes of the average fitness in a two-type population under frequency dependent selection above. Under constant selection, the average fitness will increase with probability *θ* and decrease with probability 0. In the same way as for frequency dependent selection, it will remain constant with probability 1 −* θ*. Figure 1 illustrates these results and compares frequency dependent selection to constant selection for all values of *θ*. For frequency dependent selection, there is an intersection point *θ*_∗_, where the probability to increase the average fitness and to decrease the average fitness are equal. Using Eq. (10) and Eq. (13), this becomes $2\theta_{*}^{2}-\theta_{*}^{3}=\theta_{*}-2\theta_{*}^{2}+\theta_{*}^{3}$, and we have $\theta_{*}=\frac{\sqrt{2}-1}{\sqrt{2}}$. Small values of *θ *are typically considered to be of biological relevance. In this case, frequency dependent selection tends to decrease the average fitness: for $\theta <\frac{\sqrt{2}-1}{\sqrt{2}}$, it is more likely that the average fitness of the population is decreased by a single random frequency dependent mutation; for $\theta >\frac{\sqrt{2}-1}{\sqrt{2}}$, it is more likely that it is increased.

Frequency-dependent selection can arise from different mechanisms. In a haploid population, frequency-dependent selection is caused by the interactions of different types. In this case, the fitness of a particular type depends on the frequency of its own and other types in the population. However, in a diploid population, frequency dependent selection on alleles can arise also from the interactions of two alleles at one locus [8,28,29]. Our model can be easily extended to a diploid population in such a case, which leads to different results for the average change in fitness, see Appendix.

### Games with n types

So far, we have discussed the change of the average fitness of a population consisting of at most two types. However, when two types coexist in a stable polymorphism, an additional type can enter the population and persist. To describe the interaction of individuals in a population with more than two types, we extend the 2 × 2 payoff matrix to a *n *×* n *payoff matrix *A*, where *n* is the number of types in the population. The entry in the *i*-th row and the *j*-th column, *A*_*ij*_ represents the fitness of an *i*-type individual interacting with a *j*-type individual. The fitness of type *i* on average can be written as $W_{i}(x)=\overset{n}{\underset{j=1}{\sum }}A_{\mathrm{ij}}x_{j}$, where *j* = 1,2,3…,*n*, and *x*_*j*_ is the frequency of type *j*, such that $\overset{n}{\underset{j=1}{\sum }}x_{j}=1$.

In our model, *n* is not a fixed number. When a type goes extinct, the corresponding row and column are deleted in the payoff matrix. Thus, the value of *n* decreases by one. When a mutation occurs, one row and one column are added to describe the interactions of the mutant type and resident types, which increases the size of the payoff matrix by one. The new entries introduced by a mutation are generated based on the assumption that the interactions between the mutant type *m* and any resident type *i* are similar to those between the parent type *p* and the resident type *i*. In our case, we assume *a*_*mj *_is a random variable which is drawn from a probability density function *f*(*x*) and is larger than *a*_*pj*_ with probability *θ*.

Since the complexity of the population dynamics increases considerably with the number of types, it would be difficult to obtain the changes of the average fitness in a polymorphic population of *n *> 2 types analytically. Therefore, we use the replicator equations to simulate the dynamics of the system with several types. We start the simulation from a homogenous population. However, since we are interested in the average fitness changes and other stationary quantities averaged over a long time period, the initial number of types has no effects on the results. The time intervals are sufficiently small that at most one mutant type can appear during one time interval. The probability that a resident type *i* produces a mutant type is $\mu x_{i}W_{i}\left(x\right)/\bar{W}\left(x\right)$, where *i *= 1,2,3,…,*n*. Thus the probability that a mutant arises from a resident type *i* increases with the fitness of this type. However, for the whole population, the probability that a mutant type appears is just the mutation rate, $\overset{n}{\underset{i=1}{\sum }}\mu x_{i}W_{i}\left(x\right)/\bar{W}\left(x\right)=\mu$.

We can chose arbitrary mutation rates in our simulations. However, when the mutation rate is very high, a population might experience a new mutation when it is still in a non-equilibrium state triggered by the previous mutation. In this case, the fate of a mutant is not only driven by selection, but also by the interplay of mutations. Since we are interested in the fitness consequences of frequency dependent selection, we choose the mutation rate small enough such that a population disturbed by a mutation reaches the new equilibrium before the next mutation arises.

**Figure 2:**
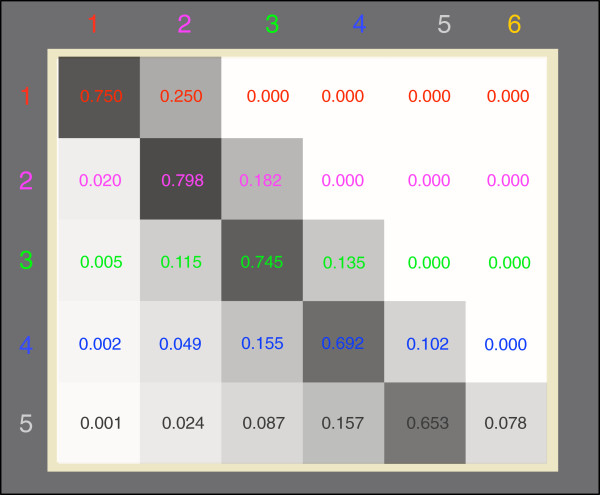
**Transition probabilities between different levels of diversity.** The entry in row *i* and column *j* is the transition probability from a stable coexistence of *i* types to a stable coexistence of *j* types, numbers are also color coded. The mutation rate is so low that the transitions between different states are caused by the appearance of a single mutation. The higher the number of coexisting types is, the more difficult the state is to be reached. Here we show the transition for up to six co-existing types (*θ *= 0.5, averages obtained over 500 independent realizations and 20000 mutations per realization).

We first look at the transition probability between different levels of diversity under mutation and selection. Once a mutation occurs it can coexist with all resident types, replace one resident type, outcompete some resident types, or go extinct. The transition matrix *T* describes this dynamics. Suppose the number of types in the current population is *n*. The element *T*_*ni *_denotes the transition probability from *n* to *i* coexisting types, where *i *= 1,2,3,…,*n* + 1, see Figure 2. We obtain the values in the transition matrix from numerical simulations. Every transition event triggered by a mutation is recorded and the probability to go from a certain number of types to another number of types is averaged over many realizations. These transition probabilities show some interesting properties. The probability to keep the current diversity (the element in the main diagonal in a row) is always higher than the probabilities to decrease or increase the diversity (all the other elements in the same row), see Figure 2 and Ref. [30]. Interestingly, for a population consisting of less than 4 types, the probability to increase the diversity *T*_*ii* + 1_ is higher than the probability to decrease the diversity $\overset{i-1}{\underset{j=1}{\sum }}T_{\mathrm{ij}}$ in the parameter regime of Figure 2. Once the population reaches the threshold of 4 types, this pattern reverses. Thus in the long run the population tends towards an intermediate level of diversity. Furthermore, we observe the ranking, *T*_12 _>* T*_23 _>* T*_34 _>* T*_45_. This suggests that the probability to reach higher levels of diversity decreases with increasing diversity even for larger number of initial types. The transition probability from one type to a two-type coexistence can be calculated analytically based on the comparison of payoff entries, see above. Thus, *T*_12 _=* p*(*a *<* c*)*p*(*b *>* d*) =* θ*/2, which is confirmed by our simulation results of *T*_12 _under different *θ *for the *n*-type model.

For a population with *n* types, the changes of the average fitness are more complicated, as the interactions between different types are much more diverse than in a two-type population. Even a classification of different types of interactions in such a population is difficult and of limited value to understand the change in average fitness. Instead, we evaluate the changes of the average fitness between these states numerically.

**Figure 3:**
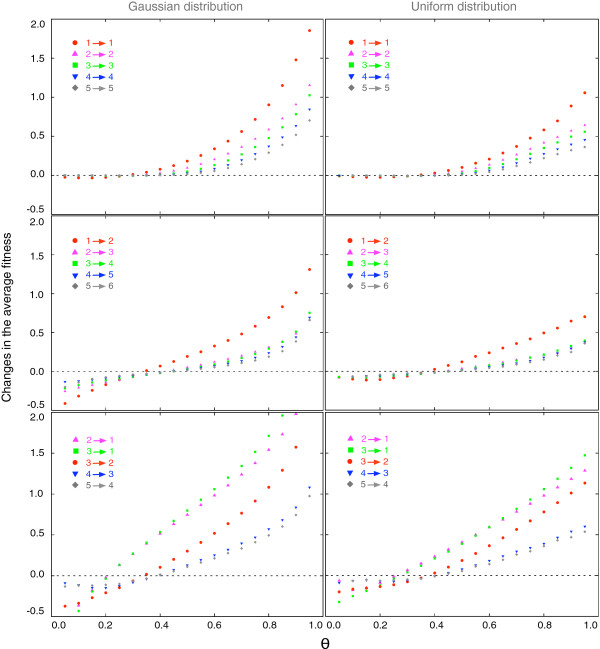
**Changes in the average fitness when a population evolves between different levels of diversity under various probabilities that a mutant payoff values is larger than the parent’s *θ*.** The symbols are simulation results based on replicator dynamics. The number of different types can either stay the same, increase by one or decrease by any number, because at most a single mutation enters the population. Note that the average fitness of the population in the new equilibrium decreases for small *θ *in all three cases after a transition. Thus even if a mutant takes over a population, the average fitness can decrease. With increasing *θ*, the average fitness will increase over time, but the fitness gain reduces with increasing diversity. The difference among results under Gaussian distribution and uniform distribution with the same variance, shows that the absolute changes of the average fitness also depends the concrete shapes of the probability distribution (every symbol is averaged over 500 independent realizations and 20000 mutations per realization. The probability distribution *f*(*x*) is Gaussian (left) or uniform (right) with variance 1).

A mutation can increase, maintain, or decrease the diversity level of the population. We present the changes of the average fitness in these three scenarios, see Figure 3, for those transitions which happen most frequently (see Figure 2). For small *θ*, mutants are more likely to obtain lower fitness than their parents type does, in the interactions with the same resident type. This can cause the decrease of the average fitness in all three situations. If *θ *is sufficiently small, the average fitness will decrease all the time. When *θ* becomes larger, the average fitness can increase. The larger *θ *is, the larger the increase is. Thus, our results under the replicator dynamics provide not only the change of the average fitness under a constant *θ*, but also the direction and magnitude of the average fitness changes. In real systems, one may expect that *θ*decreases during the adaption of the population. However, e.g. environmental changes could also increase it.

### Games with equal gains from switching

So far, we have assumed that the payoff of the mutant interacting with another resident type is derived from the payoff of its parent interacting with the same resident type. In a population with only two types, this leads to the case where the three random payoff entries, *a*, *b* and *c*, are all related to *d*. As a null model, we have assumed that *a*, *b* and *c* are uncorrelated. While this is the simplest possibility, it may not be the case for concrete biological systems. Therefore, we analyze a different case here which focuses on particular cases of frequency dependence, but includes such correlations.

We focus on an evolutionary game with the payoff matrix 

 where *ε* and *δ* are independent random variables with probability distributions *f*_*ε*_(*x*) and *f*_*δ*_(*x*) respectively. *ε* represents the effect of a mutation on the mutant type, and *δ *represents the effect of a mutation on those who interact with the mutant type. This game has the property of “equal gains from switching”, where the sum of the payoff values in the main diagonal is equal to the sum of the payoff values in the other diagonal [31]. It can arise from the assumption that the two types are close to each other in a continuous phenotype space [32]. The case of *δ *= 0 corresponds to constant selection. Note that there are no coexistence games when we assume such payoff matrices. If *ε *> 0, the mutant will take over the population (*d* + *ε* + *δ *>* d* + *δ *and *d* + *ε* >*d*), and the new average fitness becomes $\bar{W}=d+\varepsilon +\delta$. Compared with the former average fitness *d*, the average fitness increases if *ε* + *δ *> 0, and decreases if *ε* + *δ *< 0. If *ε *< 0, the mutant will be outcompeted by the wild type (*d* + *ε* + *δ *<* d* + *δ *and *d* + *ε *<* d*), and the average fitness of the population remains the same. The probability to increase the average fitness becomes $p(\bar{W}>d)=(1-\theta_{\varepsilon })\cdot 0+\theta_{\varepsilon }\cdot p(\varepsilon +\delta >0|\varepsilon >0)$, where *θ*_*ε*_ is the probability that *ε* is larger than 0, and *p*(*ε* + *δ *> 0 ∣*ε *> 0) is the conditional probability that the sum of *ε *and *δ* is larger than 0 given *ε *is larger than 0. This conditional probability can be written as

(15) $$\begin{array}{ll} \;p(\varepsilon +\delta >0|\varepsilon >0) & =\frac{p(\delta >-\varepsilon ,\varepsilon >0)}{p(\varepsilon >0)}\\ & =\frac{\int_{0}^{\infty }dx\int_{-x}^{\infty }dy\;f_{\delta }(y)\;f_{\varepsilon }(x)}{\theta_{\varepsilon }}. \end{array}$$

The values of *θ*_*ε *_and *p*(*ε* + *δ *> 0 ∣* ε *> 0), which determine the probability that the average fitness increases, depend on the concrete choice of *f*_*ε*_(*x*) and *f*_*δ*_(*x*). The integrals can only be carried out in special cases.

It is worth to mention there is a difference between games with equal gains from switching and games with independent random payoff entires on the population dynamics. In an infinite population, where genetic drift has no effect on the population dynamics, the resulting dynamics under positive frequency dependent selection and under constant selection are similar, as there are no stable coexistences. Successful mutants will invade and take over the population sequentially. The diversity will only increase if the mutation rate is high enough. On the contrary, when different kinds of interactions, especially negative frequency dependent selection, are allowed (for example, the case with independent random payoff entires), diversity can increase even for lower mutation rates (see above).

## Discussion and conclusion

Mutants with high individual fitness do not necessarily increase the average fitness of the population under frequency dependent selection. Similarly, the mutants which maximize the average fitness of a population are not necessarily those leading to a stable equilibrium in this scenario. An example for a two-type population is that a mutant interacts with the wild type in a game like the Prisoners’ Dilemma [7,26]. This is a special case of a dominance game, where the defector (the mutant) outcompetes the cooperator (the wild type) and causes a reduction in the average fitness. For example, in the RNA phage *ϕ*6, the competitive interactions among the high multiplicities-of-infection phage (the defector) and the low multiplicities-of-infection phage (the cooperator) in the same host cell are studied, which conforms to the Prisoners’ Dilemma [33]. In this experiment, when the defector invades with a low frequency, it has higher fitness than the residents (*c *>* a*), but the average fitness decreases when the defector becomes fixed (*d *>* a*).

Since natural selection works on an individual level rather than a population level, it does not always lead to an increase of the average fitness. Our random mutant games model accommodates mutations under frequency-dependent selection, which can result in an increase or decrease in the average fitness, not only for the simplest case of two types but also for an arbitrary number of mutant types. An interesting aspect of our model is that even though it allows for an infinite number of mutant types, it does not result in a continuous growth of diversity in a population, but leads to an intermediate level of diversity [30]. We assume that the payoffs are constant in time and identical for individuals of the same type. If individuals vary in their payoffs despite being of the same type, the results are altered by this additional source of randomness [34,35]. In a population with two types, we calculate a particular value *θ*_∗_, where the probability that the average fitness increases is equal to the probability it decreases. The exact value of *θ*_∗_ depends on the concrete implementation of the payoff matrix. An interesting result of our model is that the probability to decrease or increase fitness depends only on a particularly simple property of the fitness distribution. While this may not be of direct relevance to a concrete biological system, it illustrates conceptually that a decreasing fitness may not be counterintuitive even under the simplest possible assumptions of frequency dependence.

We have discussed the changes in the average fitness for an infinite asexual population under mutation and selection. Additional effects occur when the population size becomes finite and genetic drift is not negligible [30]. However, our main observation is that the average fitness at equilibrium can only increase or remain constant by random mutations under constant selection, but also decrease under frequency-dependent selection. This can shed new light on problems in evolutionary biology and leads to the exciting question on the dynamics of the average population fitness in real biological populations. In an asexual finite population, random genetic drift leads to the accumulation of deleterious mutations and an continuous decrease in the average fitness, which is well known as Muller’s ratchet [36]. Without any forms of recombination and epistasis, beneficial mutations are the only source to compensate the average fitness decline. Since the probability of increasing the average fitness by random mutations is lower under frequency-dependent selection (see Figure 1), we must conclude that asexual populations face an even bigger challenge to maintain their average fitnesses under frequency dependent selection than under constant selection in a finite population. This is particularly striking when *θ* is small, a case that is typically thought of as the biologically most relevant case.

In population genetics, the change of the average fitness has also been studied in diploid systems [37,38]. However, our approach starting from a different point of view, not only allows the interplay of mutation and selection, but also a wider interpretation of the fitness of heterzygotes. Suppose *A* and *B* are two alleles at the same locus. In population genetics, the fitness of genotype *AB* and *BA* is usually considered to be identical, which is a special case in our model called symmetric diploids. However, this does not hold in asymmetric diploids where the maternal allele and paternal allele are not equally expressed. Our model and our analysis allow both cases. In the framework of a well-mixed symmetric diploid population (corresponding to random mating), our result that the average fitness never decreases is consistent with the former statement in population genetics (see Appendix).

Frequency dependent interactions can lead to a decrease of the average fitness of a population during the process of evolution despite natural selection. This is because natural selection works on individual fitness instead of the average fitness of a population.

## Methods

We explore the population dynamics driven by random mutations under frequency dependent selection based on the replicator dynamics. For populations with only two types, we obtain analytical results by analyzing the change of average fitness between two equilibria of the replicator equations. For a population with more than two types, we simulate the evolutionary dynamics numerically. The current group of replicator equations and the current population composition determine the equilibrium that the population moves to. Once the population reaches this equilibrium, a new mutation occurs. In our model, every mutation brings a new game and consequently an additional replicator equation. Our approach corresponds to low mutation rates, where the time a population needs to reach an equilibrium is shorter than the waiting time for the next mutation.

## Appendix

### Diploid populations with two alleles

The impact of Mendelian inheritance on the population dynamics has been discussed in the framework of evolutionary game theory before [25,39-41]. In a diploid population, the combinations of two alleles at a given locus on a pair of homologous chromosomes, can be interpreted by a special two player game. Suppose there are allele *A* and allele *B*. The fitness of different genotypes, *W*_*AA*_,*W*_*AB*_ and *W*_*BB*_ can be described by a 2 × 2 matrix 

This is mathematically identical to the game with two types discussed above. Here, *W*_*AA*_ corresponds to *a*, *W*_*AB*_ to *c *=* b*, and *W*_*BB*_ to *d*. For a population initially only with homozygotes *BB*, the probability of increasing the average fitness $\bar{W}$ caused by a random new allele *A*, can be calculated by setting *c *=* b *in Eq. (10). This becomes

(16) $$\begin{array}{ll} \;p(\bar{W}>W_{\mathrm{BB}}) & =\underset{1}{\underset{︸}{p(a>d|a>b,b>d)}}\;\underset{\theta -\frac{\theta^{2}}{2}}{\underset{︸}{p(a>b,b>d)}}\\ & \;+\underset{1}{\underset{︸}{p(\frac{b^{2}-\mathrm{ad}}{2b-d-a}>d|a<b,b>d)}}\;\underset{\theta -\frac{\theta^{2}}{2}}{\underset{︸}{p(a<b,b>d)}}\\ & \;+\underset{0}{\underset{︸}{p(d>d|b<d)}}\;\underset{1-\theta }{\underset{︸}{p(b<d)}}\\ & \;=2\;\theta -\theta^{2} \end{array}$$

The probability that the average fitness decreases in such a population is 0, because the diploid *AB* and the diploid *BA* is indistinguishable, *c *=* b*. In asymmetric diploids, where the maternal alleles and paternal alleles are not equally expressed, the average fitness changes are exactly the same as shown in a general case of haploid populations.

## Competing interests

The authors declare no competing interests.

## Author’s contributions

W.H. and A.T. designed the model. W.H., B.W. and A.T. evaluated the model and wrote the manuscript. All authors read and approved the final manuscript.
